# Abusive Supervision and Employee’s Creative Performance: A Serial Mediation Model of Relational Conflict and Employee Silence

**DOI:** 10.3390/bs12050156

**Published:** 2022-05-19

**Authors:** Wang-Ro Lee, Seung-Wan Kang, Suk Bong Choi

**Affiliations:** 1 College of Global Business, Korea University, 2511 Sejong-ro, Sejong City 30019, Korea; yr2015@korea.ac.kr; 2 College of Business, Gachon University, 1342 Seongnamdaero, Sujeong-gu, Seongnam-si 13120, Korea

**Keywords:** abusive supervision, creative performance, relational conflict, employee silence, serial mediation model

## Abstract

Many previous studies on creativity have focused on discovering positive factors to improve creativity and innovation performance from leader, individual, and organizational perspectives. However, research on factors that hinder creative performance was relatively insufficient. This study examines leaders’ behavior that hinders employees’ creative performance by focusing on abusive supervision. Based on the Korean employee context, our research model draws upon constructs of abusive supervision, relational conflict, employee silence, and creative performance to hypothesize serial mediation mechanisms connecting abusive supervision to creative performance. Using survey data of 555 Korean employees, we find that abusive supervision is negatively related to creative performance. We also find that both relational conflict and employee silence mediate the relationship between abusive supervision and employee creative performance. More importantly, our empirical analysis indicates that a serial mediation effect testing a dual coordination effect was identified in the process of the leader’s abusive supervision leading to employee’s creative performance. Although many previous studies were focused on a single medium effect in the relationship between leadership types and employee creativity, this study applied the serial mediation effects in the relationship to test a dual medium effect. We further addressed a more complex process to explain the path of reducing creative performance by supervisor abusive supervision. We conclude by discussing both theoretical and practical implications.

## 1. Introduction

Prior research on creativity has focused on discovering various determining factors at the leader, employee, and organizational levels to improve innovation performance based on creativity. These studies found that a source of creativity can be divided into personal and environmental factors [1,2,3,4,5,6,7]. Personal factors include vocational ability, creative thinking, learning orientation, and creative self-efficacy, whereas environmental factors include leadership, organizational culture, and organizational support. In particular, many leadership studies have been conducted showing the positive relationship between leadership and employee creativity [1,2,3,4,5,6,7,8,9,10].

However, most of these preceding studies focused on identifying factors that improve creative performance, but they relatively lacked interest in finding factors that hinder creative performance. From this viewpoint, various studies on the negative leadership and behavior of the supervisor have been conducted such as “abusive supervision” [11], “health-endangering leaders” [12,13], “bullies” [14], “derailed leaders” [15,16], “psychopaths” [17,18], and “toxic leaders” [19,20]. Not only is an academic and systematic understanding of the supervisor’s abusive supervision necessary, but also the need to respond practically is emphasized because it acts as a negative factor for the organization. In a similar context, studies were also conducted on the destructive behavior of leaders toward organizations that deviate from organizational goals [10,19,21].

However, studies on the impact of abusive supervision on creative performance are scarce. In this vein, this study empirically analyzes the mechanism linking the relationship between abusive supervision and employee’s creative performance through relational conflict and silence behavior. Abusive supervision can be defined as “systematic and repeated behavior by a leader, supervisor or manager that violates the legitimate interest of the organization by undermining and/or sabotaging the organization’s goal, tasks, resources, and effectiveness and/or the motivation, well-being or job satisfaction of subordinates” [21] (p. 208). In this study, relational conflict and silence behavior of employees were selected as important connection variables to closely explain the process of employees’ creativity decline due to their bosses’ violence. Employees often experience opinion disagreement and conflict during their work and various activities within their organization. Moreover, this frequent conflict leads to silence within the organization, which is a more pessimistic and anti-organizational behavior. Generally, conflicts within an organization can be divided into task conflict and relational conflict. Task conflict is defined as differences in opinion among employees in task performance, and this appropriate task conflict promotes learning and positive results in organizational performance [22]. However, relational conflict is caused by individual differences, such as personality, preference, and value rather than task nature. Moreover, this relational conflict can spread to task conflict [23], eventually negatively affecting the team and organizational performance. Thus, first, this study investigates the impact of relational conflict under abusive supervision by leaders, whether it negatively affects creative performance.

Meanwhile, silence behavior can be an important obstacle hindering creative performance [24,25,26]. It is defined as a behavior of employees in an organization that intentionally does not express new ideas or information for cognitive, emotional, or behavioral reasons [26,27]. This silence behavior was reported to negatively impact creative behavior by preventing organizational learning and knowledge sharing, which are necessary factors for enhancing creative performance [25,26]. Previous studies on leadership revealed that leaders’ proper feedback behavior reduces silence behavior as employees perceive that the leader supports them and provides timely advice for compensation [26,28,29]. However, these studies neglect to explain the role of silence behavior as a key mechanism that negatively affects creative performance when performance is not linked to proper compensation due to abusive supervision. In addition, previous studies have focused on analyzing the simple mediation process in the relationship between abusive leadership and organizational performance. For example, abusive behavior of leaders was found to hurt job satisfaction [11] and organizational commitment [30], and increase turnover intention [11], deviant behavior [31], and stress [32]. These past studies have lacked a more logical and detailed explanation of the causal relationship between abusive supervision and creative performance [8,9,10]. Therefore, this study strengthened the explanation of the causal relationship in the process of abusive supervision to employees’ creative performance through a serial mediation model of relational conflict and silence behavior.

To accomplish this, first, we examined the effect of abusive supervision on creative performance. Second, we investigated the mediation effect of both relational conflict and silence behavior in the relationship between abusive supervision and employees’ creative performance. Finally, this study explored a dual coordination process that negatively affects creative performance based on a serial mediation model of relational conflict and employee silence.

## 2. Theoretical Background and Hypotheses

### 2.1. Abusive Supervision and Employee’s Creative Performance

According to Tepper (2000), abusive supervision is the “systematic and repetitive behavior exerted on a subordinate through verbal and non-verbal behavior excluding physical contact of the supervisor [11] (p. 178). For example, it refers to actions such as a boss mocking his subordinates, violating the privacy, failing to keep his promises, or repeatedly lying to his subordinates [33]. Such impersonal supervision was found to cause psychological pain to individuals and adversely affect organizational performance [33]. Previous studies have shown that supervisor’s abusive supervision increases aggression and deviant behavior [31], turnover intention [11], excessive drinking [34], and stress [32]. It also decreases job satisfaction [11], organizational satisfaction [11], and organizational citizenship behavior [35,36].

In this context, we assume that organizational creativity must be understood through the interaction between individuals and organizations, and creativity means the basis for members to successfully implement new ideas [37]. Moreover, it is the source for creating new products and services [38]. In fact, given that abuse supervision has various negative effects on employee performance, job satisfaction, positive self-evaluation, well-being, and stress [11,39], abusive supervision is expected to negatively affect employee creative performance in the following ways. First, according to the resource conservation theory [40], abusive supervision can create stress for the employees and negatively affect job performance. In other words, if the supervisors repeatedly ridicule, neglect, and blame his or her subordinates, the recipients of such behavior can experience emotional exhaustion, loss of resources, and stress [11]. These negative emotions can negatively affect creativity which requires high concentration and flexibility.

Second, employees’ intrinsic motivation is an essential component of creativity [41]. If the supervisor does not acknowledge or ignores the employees’ efforts, their intrinsic motivation for work decreases. This can, in turn, decrease creativity. In other words, the abusive supervision is expected to negatively affect creative performance by hindering positive energy, resilience, active participation, and job commitment [42,43]. Third, leader feedback as an environmental antecedent has an essential influence on creativity [44,45]. Employees exposed to impersonal treatment by their supervisor, such as lying or not keeping promises, are less engaged in the effort and creative behavior toward their job responsibilities [8]. Therefore, the following hypothesis is established on the basis of the earlier logic.

**Hypothesis** **1.** *The abusive supervision negatively affects employees’ creative performance.*

### 2.2. Mediating Effect of Employee Relational Conflict

In previous studies, conflict within an organization was considered the cause of unnecessary confusion and energy dissipation, resulting in negative consequences for the organization’s decision making and performance [46,47,48,49]. Meanwhile, some studies argued that very low amounts of conflict within the organization leads to poor performance due to complacency [23,50]. Therefore, the need for conflict management within the organization was raised to promote problem solving and positively affect the organization by properly managing conflicts.

Organizational conflict can be divided into task conflict and relationship conflict [47,51]. Task conflict refers to differences in opinions such as perspectives, decision-making matters, and work procedures in performing tasks. Meanwhile, relationship conflict refers to inconsistencies arising from personal problems, such as personality, preference, value, and attitude. Relational conflict mainly appears as negative emotions, such as anger, distrust, threats, and disappointment [47,50]. Previous studies have reported that relational conflict negatively affects the organization’s productivity, job satisfaction, and turnover intention [52,53,54,55]. These results suggest the necessity of research on impersonal supervision of the boss and the important influence of the relationship conflict between the supervisor and the subordinate due to abusive supervision on creative performance.

In this study, relational conflict is expected to negatively mediate the relationship between the supervisor’s abusive supervision and the employee’s creative performance for the following reasons.

First, subordinates who have experienced high relational conflicts are likely to reduce creative behavior by using energy and time to perform tasks and resolve emotional conflicts [56]. According to the resource conservation theory, subordinates who experience their supervisor continuously mentioning their mistakes in front of others or having a supervisor venting stress will cause them to experience increased relational stress with the supervisor. Hence, it can negatively affect creative activities by exhausting time and passion for thinking about changing jobs or focusing on tasks to relieve stress.

Second, relational conflicts arise when employees are ignored and not recognized by their bosses for their efforts, thus reducing creative behavior [56,57]. When employees present excellent creative ideas for solving work problems, employees who are unconditionally criticized and ignored by their bosses rather than receive rational criticism or alternatives may reduce their willingness to propose new ideas to produce creative results.

Third, relational conflict causes job conflict that negatively affects team performance by limiting the flow of information that promotes interaction between organizational members [58]. Additionally, members of the organization who have experienced abusive supervision are more likely to communicate their aggression and hostility directly to other coworkers [31]. In this way, relational conflict with the supervisor causes job conflict and simultaneously worsens relationship conflict between other subordinates, hindering mutual learning and communication between subordinates. In the end, these negative interaction effects between employees can be expected to affect creative performance negatively.

The increase in relational conflict due to the supervisor’s abusive supervision causes stress, exhausting the time and energy to be used for problem analysis and alternative presentation. Additionally, it is expected to interfere with organizational learning and knowledge sharing through interaction and communication between subordinates for creative problem solving. Therefore, we expect mechanisms that negatively affect creative performance by lowering voluntary participation and execution. Based on the earlier logic, the following hypothesis is established.

**Hypothesis** **2.** *Relational conflicts negatively mediate the relationship between the supervisor’s abusive supervision and creative performance.*

### 2.3. Mediating Effect of Employee Silence Behavior

Experiencing abusive supervision of the supervisor or authoritarian organizational culture can cause silence behavior by shrinking the words and actions of employees. Silence behavior is divided into defensive silence and resignation silence according to the actor’s motivation. Defensive silence is an act of silence that employees do not express due to criticism of their supervisor and fear of poor reputation when presenting ideas, information, and opinions. Meanwhile, resignation silence is defined as a silence action that does not externally express proposals beneficial to the organization due to employees’ disregard for good idea suggestions, efforts, and performance [27].

However, Pinder and Harlos (2001) mentioned that the silence of employees is an act that recognizes the need to improve the organizational situation but intentionally expresses it for cognitive and emotional reasons [26]. Van Dyne et al. (2003) indicated that differences in status within the organization, concerns about the negative stigma after presenting opinions, and concerns about interpersonal damage after presenting dissenting opinions, are the main source of employees’ silence [25,27,59].

Silence behavior within an organization presents as intentionally hiding creative ideas and valuable suggestions related to work performance, reducing various role activities that improve the organization’s competitiveness [25,27,60]. Additionally, since silence behavior restricts open discussion, it may negatively impact active communication and organizational learning, eventually making it difficult to exert creativity within the organization [61]. This atmosphere of silence leads to inefficiency in organizational change and reduces the internal motivation of members [25]. As a result, it suggests that the subordinate’s silence behavior will negatively affect the employee’s creative performance.

Therefore, the behavior of silence negatively mediates the relationship between supervisor’s abusive supervision and creative performance for the following reasons. First, if the supervisor repeatedly ignores the employee’s job performance, the employee forgoes finding a new problem-solving method or active expression of opinion to find creative ways to complete their task, which increases silence behavior [27]. Ultimately, when employees perceive that mental and material rewards are not linked to performance, it decreases voluntary participation and negatively affects creative performance.

Second, to avoid emotional damage or uncomfortable situations caused by abusive supervision, such as invasion of privacy by the supervisors, employees tend to maintain a distance from the supervisor by choosing silence behavior [62]. According to several studies, this negatively affects the creative performance of the organization by blocking open discussions, knowledge sharing, and mutual learning with the supervisor [25,61]. Hence, the increase in silence behavior hinders interaction with the supervisor and knowledge sharing, negatively affecting creative performance. Third, employees who have experienced degradation to their reputation in front of their colleagues can expect to act with silence behavior and reduce creative performance by not voluntarily participating in their jobs [27,63,64]. In other words, to avoid deteriorating one’s reputation in the interaction between the supervisor and its members, employees will intentionally increase silence behavior, which will negatively affect creative performance. Based on the above logic, the following hypothesis is established.

**Hypothesis** **3.** *Employee silence behavior negatively mediates the relationship between abusive supervision and creative performance.*

### 2.4. Sequential Mediating Role of Relational Conflict and Silence Behavior in the Relationship between Abusive Supervision and Creative Performance

Previous studies have argued that to resolve relational conflicts caused by abusive supervision, employees must show a pattern of behavior that avoids disputes in face-to-face or discussion with their supervisors rather than directly communicating with them [65,66,67]. Under these circumstances, supervisors are likely to use compulsory methods on employees to manage relational conflicts [68]. Employees experiencing relational conflicts caused by their supervisors’ abusive supervision avoid unfavorable reputation or personnel disadvantages from their supervisors. They also think that their supervisors will be indifferent to their proposals or job performance, leading to defensive silence actions [27,69,70].

Additionally, this silence behavior of employees reduces active participation as a phenomenon that occurs when negative internal evaluation or unfairness is recognized. Therefore, creative proposals are deliberately suppressed and negatively affect organizational reform and learning. Consequently, it also negatively affects creative activities within the organization [25,60,61]. These results suggest that abusive supervision causes relational conflicts within the organization, leading to silence behavior of employees and ultimately to a series of negative effects on employee creative performance.

Therefore, relational conflict and employee silence are expected to continuously and negatively mediate the relationship between abusive supervision and creative performance for the following reasons. In other words, we assume that abusive supervision is expected to increase relational conflict within the organization, increase the silence of employees, and eventually reduce employees’ creative performance.

First, when job performance and compensation are not properly linked, which is often caused by abusive supervision, the supervisor’s disregard for the employee’s job performance forms a state of tension between members, leading to emotional conflict. Moreover, employees increase their silence behavior without making job improvement remarks [71]. In turn, this silence behavior, which has a tone of resignation and defense by employees, will negatively affect creative performance by preventing voluntary motivation to participate in work.

Second, when a subordinate witnesses a supervisor’s behavior that degrades employees’ reputation through public criticism or gossiping against a coworker, it causes a relational conflict between members of the team. Thus, the employees are afraid of a negative reputation from their supervisor and colleagues when proposing creative ideas. In turn, this hinders knowledge sharing, work learning, and cooperative activities, eventually negatively affecting creative performance [72].

Third, the supervisor’s act of ignoring the employee’s efforts for job improvement increases emotional conflict between members. Accordingly, employees who have not been encouraged and supported by their supervisors increase resignation and silence behavior which leads to actions that forgo new attempts which can negatively affect creative performance [73]. In sum, the supervisor’s disregard for employees’ efforts for improving job performance and the supervisor’s public criticism of fellow employees increases friction, tension, and emotional conflict among members. It also reduces employees’ willingness to participate voluntarily. Therefore, we assume that employees’ relational conflict and silence behavior negatively and continuously mediates the relationship between abusive supervision and creative performance. Based on this discussion, the following hypothesis is established.

**Hypothesis** **4.** *Relational conflict and silence behavior of employees serially mediate the relationship between abusive supervision and creative performance, such that abusive supervision sleep undermines relational conflict, which in turn increases silence behavior, and finally, increased silence behavior reduces creative performance.*

**Figure 1:**
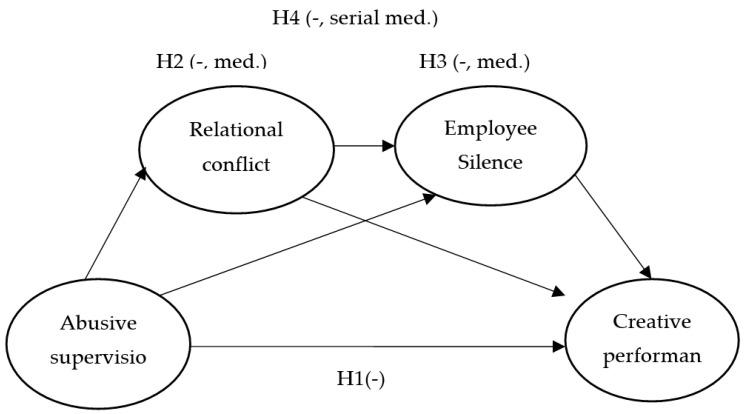
The theoretical research model (made by author, 2022).

The theoretical model of this study is depicted in Figure 1.

## 3. Methodology

### 3.1. Sample and Procedure

The empirical analysis of this study adopted a nonrandom sampling method considering the specific characteristics of employee perception of key variables used in this study, such as abusive supervision, relational conflict, and silence behavior. Following suggestions from previous studies using this non-probability method [74], we could more effectively select sample groups based on our judgment and preference for our research objectives. Specifically, we adopted the purposive sample method which could give the most relevant and plentiful data. Our key variables include the leader’s negative style and the employee’s counterproductive behavior such as relational conflict and employee silence. Since leadership styles appear in various types, we needed to choose the respondents who suffered from abusive supervision. Moreover, the purposive sample method was needed to explain employees’ psychological status after experiencing abusive supervision. We were careful in selecting samples because we assumed it was difficult for people to publicly criticize their leaders and respond to their unproductive behavior. Thus, we also tried to ensure anonymity.

In the first step, we contacted the HR managers to explain the purpose of the survey. In the second step, when we obtained their permission, we requested to ensure anonymity and consider the diversity of samplings such as gender, tenure, and age. Next, we distributed anonymous online and offline questionnaires to employees. Participants were explained through the purpose and procedures of the survey and the benefits and disadvantages that may arise from participating. Moreover, participants were offered the freedom to withdraw from the survey at any time.

Of the 700 distributed questionnaires, 555 were returned, that is, a response rate of 79%. Five hundred fifty-five questionnaires were used for the final analysis, excluding the questionnaires with invalid or no response. Among the 555 respondents of this study, 90.5% were male. The average age of the respondents was 39.9 years (SD = 8.02), and the average duration of regular education was about 15.3 years (SD = 1.78).

### 3.2. Measures

We analyzed all the variables at the individual level. Unless indicated, the study variables were measured using a 5-point Likert scale (1 = strongly disagree, 5 = strongly agree). We also assessed internal reliability using Cronbach’s alpha values.

#### 3.2.1. Abusive Supervision

We used 15 items adopted by Tepper (2000) to measure abusive supervision [11]. This measurement was also used in the context of the Korean workplace in the previous study [75,76]. Sample items are “My boss treats me like a mockery”, “My boss doesn’t recognize that I put in a lot of effort and hard work”, and “My boss prevents me from interacting with my colleagues”. The Cronbach’s α of our scale was 0.963.

#### 3.2.2. Relational Conflict

We used four items adopted from Jehn (1995) to measure relational conflict [47]. Previous studies for Korean workers [77,78] also used this measurement. The sample items are “Our team tends to have emotional conflicts between members”, “Our team tends to have personality conflicts between members”, and “Our team tends to have a tense relationship between members”. The Cronbach’s α of our scale was 0.884.

#### 3.2.3. Silence Behavior

We used ten items adopted from Van Dyne et al. (2003) to measure employee’s silence behavior [27]. As previous studies have used this measurement for Korean employees [79,80], we confirm the validity of measurement. The sample items are “I’m worried about how my boss will react, so sometimes I have ideas for organizational change, but I don’t suggest them”, and “Sometimes I don’t offer suggestions to my boss for improvement because I don’t think talking can change the situation”. The Cronbach’s α of our scale was 0.952.

#### 3.2.4. Creative Performance

We used 13 items adopted from Zhou and George (2001) to measure creative performance [37]. This measurement was also used in the context of the Korean workplace in previous study [76].The sample items are “I propose a new way to achieve a goal”, “I am not afraid to take risks”, and “I often have a new approach to a problem that I did not have before”. The Cronbach’s α of our scale was 0.958.

#### 3.2.5. Control Variables

According to previous studies, age, and education period affected supervisor’s abusive supervision and individual creativity [41,81,82,83]. Therefore, demographic variables, such as age, and education period were included as control variables in the empirical analysis of this study. Age was measured in years. The level of education was measured as the year of regular education completed by respondents.

#### 3.2.6. Analytical Approach

We conducted the factor analysis and the correlation analysis using the programs SPSS 21.0 and AMOS 21.0 to analyze the study sample. We used the PROCESS-macromodel by Hayes (2013) and bootstrapped 10,000 times to analyze the double mediation model. An analysis method suggests the interaction effect and indirect effect as a confidence interval when analyzing the interaction effect and the indirect effect [84]. Then, we conducted a confirmatory factor analysis (CFA) to evaluate the construct validity of the measures of the variables. In addition, convergence and discriminant validity were verified using construct reliability (CR) and average variance extracted (AVE) values to confirm the degree of convergence of constructs. According to the criteria presented by Fornell and Larcker (1981) [85], all values of Cronbach α, the reliability coefficient, were above 0.7. Moreover, all CR values were 0.884 or higher. As all constructs exceeded the threshold AVE value of >0.50, it is concluded that they could measure the latent variables. Hence, they fulfilled the convergent validity criteria.

## 4. Result

### 4.1. Descriptive Statistics

We conducted a correlation analysis between the latent variables of the measurement model. As a result, the expected correlations for six variables were sufficient (see Table 1), so we conducted the structural model analysis, the final research model. The results of the variation inflation factor (VIF) analysis revealed that all VIF values were less than 2.0 (less than the standard 10), thus verifying the multicollinearity problem between some highly correlated variables.

### 4.2. Measurement Model

We conducted the measurement model comparison analysis to test the model fit of the hypothetical model and tested four alternative models. Table 2 presents the results of the hypothesized three-factor model. The goodness-of-fit indicators met the strict cut-off points: χ^2^ = 1716.90; df = 762, *p* < 0.001; χ^2^/df = 2.25; CFI = 0.96; TLI = 0.95; RMSEA = 0.05. (*p* < 0.001). Meanwhile, the factor loadings ranged from 0.57 to 0.91 at a statistically significant level (*p* < 0.001). Four alternative models were tested [86]. The results noted that the hypothesized model differs from the alternative models and presents the best-fit indicators [86].

### 4.3. Hypothesis Testing

We used the serial multiple mediation model by Hayes (2013) to test how the variables used in this study interact with each other [84]. We conducted bootstrapping analysis using 10,000 bootstrapped samples to test the indirect effect of the mediation and serial mediation hypotheses. If the upper and lower limits of the coefficient obtained in the 95% confidence interval (IC) do not include “0”, it can be determined as a significant value. Table 3 shows the results of path analysis for hypothesis testing. Looking at the path analysis results, first, we analyzed that abusive supervision had a direct negative effect on creative performance (β = −0.16, *p* < *0*.01), supporting hypothesis 1. Additionally, results of bootstrapping method from Table 3 revealed a negative mediating effect (β = −0.07, CI: −0.12 to −0.02) on abusive supervision → relation conflict → creative performance. Hypothesis 2 was also supported by the analysis. Meanwhile, a negative mediating effect (β = −0.12, CI: −0.17 to −0.07) on abusive supervision → employee silence behavior → creative performance was also determined, thereby supporting Hypothesis 3. In addition, the sequential continuous negative mediating effect (β = −0.03, CI: −0.06 to −0.02) from the abusive supervision → relation conflict → employee silence behavior → creative performance did not include “0” in the 95% confidence level. Relational conflict and employee silence mediated the relationship between supervisor’s abusive supervision and creative performance successively and sequentially. The serial mediation hypothesis 4 was also supported, stating that the supervisor’s abusive supervision increases relation to conflict and increases employee silence, which in turn reduces creative performance.

## 5. Discussion and Implications

### 5.1. Theoretical Implications

This study focused on the behavioral perspective of members of the organization, which is the starting point and subject of creative performance, in that creative performance is an essential element of corporate survival. From this perspective, we analyzed how supervisor’s abusive supervision hindered creative performance in an organization. More specifically, the framework explains the complex process that hinders the creative performance of members by abusive supervision that was suggested and tested in the context of Korean workers. Results of the study reveal that the abusive supervision in the organization negatively affects the employee creative performance by inducing relational conflict between members of the organization, silence behavior of employees, and dual coordination effects of them.

The theoretical implications of the results of this study are as follows. First, this study expanded the results of the existing research by first confirming the mediating effects of each relational conflict and employee’s silence for the boss’ impersonal supervision to understand the various processes leading to employee creativity. Many past studies on creativity have focused on discovering the determinants of creativity at the behavior and type of leadership, individual, and organizational level factors [1,2,3,4,5,6,7]. However, studies on factors that hinder creativity were relatively insufficient [8,9,10]. This research extends the creativity literature by investigating the explanatory mechanisms of abusive supervision from a cognitive and behavioral perspective. Moreover, this study indicates that relational conflict and silence behavior are processes that underlie some of the main effects of abusive leadership on creative performance.

This study is meaningful in that it focuses on the effect of abusive supervision, identifies supervisors’ behavior that hinders employee’s creative performance, and addresses detailed paths for impersonal supervision to harm creative performance.

Second, previous studies that examined the negative effect of abusive supervision on various performances at individual and organizational levels, such as psychological stress [32], turnover intention [11], and deviant behavior [31], have focused mainly on the direct effect and the bi-directional relationship between them.

Using a serial mediation model, this study found a sequential double mediation effect by simultaneously inputting relational conflict and silence behavior in the relationship between abusive supervision and creative performance. Previous studies did not cover these negative effects of relational conflict and silence behavior in relation to creative performance with abusive supervision. More importantly, through this double mediation effect verification, we confirmed a series of interconnected mediating effects and the individual effects of employee silence and relationship conflict, which is expected to lead to the expansion of existing leadership and creativity studies.

Third, based on existing resource conservation theory [40], the applicability of this theory was expanded to creativity research by proving that the supervisor’s impersonal supervision causes relationship conflict and silence behavior.

### 5.2. Practical Implications

Based on the results of the empirical analysis, the practical implications of this study are as follows. First, the abusive supervision of the boss was found to hurt the creative performance of the employee. Recently, it has been made clear that there is an increase in employee stress due to supervisors’ power abuse [32], an increase in turnover intention [11], and a decrease in organizational performance [11,31]. Already, Sweden, Canada, France, and Korea have enacted the “Work Bullying Prohibition Act”. Therefore, the organization should reorganize continuous supervisor-level education and workplace rules of conduct to prevent abusive supervision. Additionally, convenience must be increased by opening an online and offline reporting center in the company so that reports can be made anytime and anywhere in the event of abusive supervision and workplace harassment.

Workplace harassment due to abusive supervision includes physical and mental pain beyond the appropriate range of work by using the superiority in the status relationship. Therefore, an organization should also strive to improve the organizational culture such that if an act of abusive supervision occurs in the workplace, it may be punished.

Second, relationship conflict has a mediating effect between the supervisor’s impersonal supervision and creative performance. These findings are inferred to amplify relationship conflicts by increasing mental stress and tension among employees under abusive supervision from their supervisors who influence employees’ personnel rights, performance evaluation, and reputation within the organization. Therefore, the organization needs an appropriate conflict management system within the organization, such as reducing the supervisor’s abusive supervisory behavior by conducting multi-faceted evaluation and regular monitoring of the supervisor, while securing time to listen and consult with subordinates.

Third, silence behavior also had a negative mediating effect between the supervisor’s abusive supervision and creative performance. Therefore, the supervisor needs to form trusting relationships with employees so that they do not have personal feelings or prejudices when evaluating employees’ performance or behavior. Additionally, this study suggests the necessity to minimize the silence behavior of employees by creating a peaceful atmosphere to promote expression. Thus, to enhance the creative performance of employees, the organization must introduce a proper education and training system within the organization that manages supervisor’s abusive supervision, relational conflict, and silence behavior of employees, and a manager’s willingness to continue to practice them as a culture.

### 5.3. Limitations and Direction for Future Research

The following limitations are mentioned to suggest the direction of future research. First, the data used in the empirical analysis of this study were collected simultaneously using the questionnaire response method. Therefore, in future studies, a method for reducing common method bias, such as separating the response time and response source, is suggested. Second, the data used in this study were collected from Korean workers in various workplaces. However, in future studies, more meaningful research results will be obtained if research samples are expanded to other countries, industries, and businesses. Third, this study used cross-sectional data based on a certain point in time, which is insufficient to grasp a more accurate causal relationship. In future studies, a longitudinal research design is needed to confirm the findings of the research. Finally, in this study, sequential double mediation effects were identified by setting parameters, such as relational conflict and silence behavior, which are intrinsic motivational factors affecting the supervisor’s abusive supervision. However, if mediating and moderating variables are explored and used at the team and organizational levels, conditions leading to creative performance from the supervisor’s abusive supervision would be made clearer. In other words, more meaningful implications will be derived if multilevel analysis, including organization and team-level factors, is performed.

## Figures and Tables

**Table 1:** Descriptive Statistics and Inter-correlations.

	Mean	SD	1	2	3	4	5	6
1. Age	39.88	8.02	-					
2. Education	15.25	1.78	−0.04	-				
3. Abusive supervision	1.57	1.48	0.12 **	−0.06	(0.96)			
4. Relational conflict	2.08	0.72	0.26 **	−0.04	0.43 **	(0.88)		
5. Employee silence	2.00	0.70	0.05	0.01	0.53 **	0.45 **	(0.95)	
6. Creative performance	3.42	0.65	0.25 **	0.07	−0.30 **	−0.24 **	−0.37 **	(0.96)

Note: N = 555. Cronbach’s alpha values are reported in parentheses on the diagonals. ** *p* < 0.01 (two-tailed).

**Table 2:** Model Fit Statistics for Measurement Models.

Measurement Model	Χ^2^	df	CFI	TLI	RMSEA	Δχ^2^	Δdf
Baseline (hypothesized) four-factor model	1716.90	762	0.96	0.95	0.05		
Alternative 1 (three-factor model) ^1^	2737.29	765	0.91	0.90	0.07	1020.39 **	3
Alternative 2 (two-factor model) ^2^	4356.48	767	0.83	0.81	0.09	2639.59 **	5
Alternative 3 (two-factor model) ^3^	4984.47	767	0.80	0.78	0.10	3267.57 **	5
Alternative 4 (one-factor model) ^4^	7864.82	768	0.88	0.81	0.17	6147.92 **	6

Note: N = 555. ** *p* < 0.01 (two-tailed test). ^1^ Three-factor model evaluating abusive supervision, relational conflict, and employee silence on the same factor. ^2^ Two-factor model with abusive supervision and relational conflict and silence behavior on the same factor. ^3^ Two-factor model with abusive supervision, relational conflict, and silence behavior on the same factor. ^4^ One-factor model with abusive supervision and relation conflict and silence behavior, creative performance on the same factor.

**Table 3:** Results of Regression Analyses and Bootstrapped Indirect Effect Test.

Main Effects	Relation Conflict	Employee Silence	Creative Performance
Age	0.02 ***	−0.01 *	0.03 ***
Education	−0.003	0.02	0.03 *
Abusive supervision (AS)	0.52 ***	0.53 ***	−0.16 **
Relational conflict (RC)		0.28 ***	−0.13 ***
Employee silence (ES)			−0.23 ***
F	53.8 ***	74.66 ***	36.77 ***
R^2^	0.23	0.35	0.25
Indirect Effects	Estimate	Lower Level	Upper Level
AS→RC→CP AS→ES→CP AS→RC→ES→CP	−0.07 −0.12 −0.03	−0.12 −0.17 −0.06	−0.02 −0.07 −0.02

Note: N = 555. The indirect effect estimated was tested for significance using 95% bias-corrected bootstrapped. confidence intervals. * *p* < 0.05, ** *p* < 0.01, *** *p* < 0.001 (two-tailed); bootstrap resampling = 10,000.

## Data Availability

Not applicable.
